# Bifunctional catalytic activity of anion-doped LaSrCoO_4_ for oxygen reduction and evolution reactions

**DOI:** 10.1098/rsos.240387

**Published:** 2024-10-09

**Authors:** Ittoku Nozawa, Hidehisa Hagiwara

**Affiliations:** ^1^ Hydrogen Isotope Research Center Organization for Promotion of Research, University of Toyama, 3190 Gofuku, Toyama 930-8555, Japan

**Keywords:** perovskite-related compounds, bifunctional catalyst, oxygen reduction reaction, oxygen evolution reaction, mixed-anion compounds

## Abstract

Here, we synthesized Co-based, anion-incorporated‍ ‌R‌u‌d‌d‌l‌e‌s­d‌e‌n‌–‌Popper perovskite electrocatalysts (LaSrCoO_4*−x*_X_*y*_) and compared their catalytic performances in the oxygen reduction reaction (ORR) and oxygen evolution reaction (OER). The ORR mechanism with the newly synthesized F-doped LaSrCoO_4_ catalyst was dominated by a four-electron process, and the number of electrons involved in the reaction increased compared with that for LaSrCoO_4_. The OER activity of the hydride-doped LaSrCoO_4_ catalyst was the highest among the LaSrCoO_4_ system catalysts. Density functional theory calculations revealed that there is a correlation between the Co 3d unoccupied orbital band centre and the OER activity. The addition of anions and substitution of metal sites improved the ORR and OER activities of the catalysts. Our findings confirmed that the addition of heteroatom anions can improve the activity of perovskite-type electrocatalysts, promoting their application in various fields.

## 1. Introduction

Recently, to solve the growing global energy problems, researchers have attempted to improve the efficiency and durability of energy-conversion devices, such as fuel cells, metal–air batteries, and water electrolysis cells [1–8]. The oxygen reduction reaction (ORR, O_2_ + 2H_2_O + 4e^−^ → 4OH^−^ under alkaline conditions) and oxygen evolution reaction (OER, 4OH^−^ → O_2_ + 2H_2_O + 4e^−^ under alkaline conditions) are the main chemical reactions occurring in these devices. Presently, perovskite-type composite metal oxides incorporating transition metals (ABO_3_) are known to exhibit ORR and OER activities [9] and show potential for use as low-cost, high-performance catalysts. The ORR and OER activities of catalysts are closely related to the electronic state of their B-site cations [10,11], the relationship between the O 2p-band centre and the Fermi level [12], the charge-transfer energy [13], the metal–metal average bond distance [14] and the metal–oxygen bond distance [15]. Regarding the electronic state of the B-site cation, the ORR activity of ABO_3_-type perovskites is high when the number of e_g_ electrons in the 3d orbital is in the range of 0.8−1.0. For the OER, Ba_0.5_Sr_0.5_Co_0.8_Fe_0.2_O_3_ (BSCF) with 1.2 e_g_ electrons in the 3d orbital exhibits the highest activity, and consequently it has received significant research interest [16,17]. Density functional theory (DFT) studies of this catalyst have also been conducted. The OER activity of a catalyst with different A-site cations, i.e. (Ln_0.5_Ba_0.5_)CoO_3-*δ*_ (Ln = Pr, Sm, Gd, Ho), has been determined to improve as the O 2p-band centre approaches the Fermi level [12]. Similarly, using DFT calculations, the OER activities of SrBO_3_ (B = Ti, V, Cr, Mn, Fe, Co), YBO_3_ (B = V, Cr, Mn, Fe, Co, Ni) and LaBO_3_ (B = V, Cr, Mn, Ni) were determined to increase as the charge-transfer energy decreased. The charge-transfer energy refers to the gap between the O 2p-band centre and the 3d unoccupied orbital band centre of the B-site cation. A variety of perovskite oxides have been studied using various methods, as described above. These are mainly perovskite oxides incorporating various metal cations at the A and B sites. Another type is anion-incorporated perovskites, which contain a heteroatom at the anion site (ABO_3-*x*_X_*y*_). For example, BaTiO_2.4_H_0.6_ [18], La_1-*x*_Sr_*x*_FeO_3-*y*_F_*y*_ [19] and SrCrO_2_H [20], which exhibit low catalytic activities, are not suitable for mass production because they require a high temperature and pressure of 1000℃ and 5 GPa, respectively, for synthesis. Therefore, Ruddlesden–Popper-type layered perovskites (general formula = A_*n*+1_B_*n*_O_3*n*+1_), i.e. A_2_BO_4_ (*n* = 1) [21,22], A_3_B_2_O_7_ (*n* = 2) [23] and A_4_B_3_O_10_ (*n* = 3) [24], as well as a Ruddlesden–Popper-type layered perovskite with an added anion, have been investigated [14,19,25]. In addition, Ruddlesden–Popper-type layered perovskites with anion addition and high OER activity have been reported [25]. However, few studies have elucidated in detail the relationship between anion addition and the catalytic activity.

In this study, attempts were made to introduce H^−^ and then F^−^ into LaSrCoO_4_, a Ruddlesden–Popper-type layered perovskite, and the ORR and OER activities of the resulting catalyst were evaluated. To the best of our knowledge, the electrocatalytic activity of Co-based Ruddlesden–Popper layered perovskites co-doped with hydride and fluorine has not previously been investigated. We examined the relationship between the electrocatalytic activity and the electronic state and band structure of the catalyst’s B-site cation, and the stability of the anion-doped catalyst for electrochemical reactions.

## 2. Methods

### 2.1. Catalyst preparation

#### 2.1.1. Synthesis of LaSrCoO_4_

Five grams of LaSrCoO_4_ was synthesized using the polymerized complex method. Ethylene glycol (99.5%, 4.5 g, citric acid (98.0%, 6.7 g) and stoichiometric amounts of La(NO_3_)_3_·6H_2_O (99.9%), Sr(NO_3_)_2_ (98.0−102%) and Co(NO_3_)_2_·6H_2_O (99.5%) ( all from FUJIFILM Wako Pure Chemical Corp.) were added to a 300 ml beaker and heated at 280°C while stirring until the contents solidified. The resultant mixture was pretreated using a mantle heater at 350°C for approximately 2 h and subsequently calcined at 950°C for 5 h under flowing O_2_ (50 ml min^−1^).

#### 2.1.2. Synthesis of hydride-doped LaSrCoO_4_ (LaSrCoO_4_-H)

The oxyhydride was synthesized using CaH_2_, following the method of Hayward *et al*. [26]. LaSrCoO_4_ (3.0 g) was dried at 200°C under vacuum, and two equivalents of CaH_2_ (95.0%, FUJIFILM Wako Pure Chemical Corp.) were mixed in a glove box under an Ar atmosphere. Thereafter, the powder was flame-sealed in a silica tube. The tube was heated at 450°C for 90 h. The product was washed with 0.1 M NH_4_Cl (99.5%, FUJIFILM Wako Pure Chemical Corp.) in methanol (99.8%, FUJIFILM Wako Pure Chemical Corp.) to remove excess CaH_2_ and CaO. Finally, the powder was obtained by washing it with pure water in conjunction with centrifugation.

#### 2.1.3. Fluorination of LaSrCoO_4_-H with NH_4_F (LaSrCoO_4_-F)

LaSrCoO_4_-H (0.4 g) dried at 70°C under vacuum and NH_4_F (97.0%, 4.0 mg, Kanto Chemical Co.) were mixed in a glove box under an Ar atmosphere. Afterwards, the powder was transferred to a polytetrafluoroethylene container, sealed in an autoclave and heated at 150°C for 24 h.

### 2.2. Characterization

The electronic state of the sample (i.e. chemical bonding state and oxidation state) was assessed by X-ray photoelectron spectroscopy (XPS; Thermo Fisher Scientific ESCALAB250Xi). Thermogravimetry–differential thermal analysis (TG–DTA) was conducted using a ThermoPlus2 (Rigaku Corp.) at a heating rate of 2°C min^−1^ under flowing air (100 ml min^−1^). The surface state and particle diameter of the powders were observed by field-emission scanning electron microscopy (FE-SEM; JSM-6701, JEOL). The specific surface area was measured using a gas-adsorption measuring device (BELSORP-mini II, MicrotracBEL Corp.). The crystal structure of the powders was determined by X-ray diffraction analysis (XRD; X’Pert-Pro-mpdpw3040) using Cu Kα (*λ* = 1.5418 Å) radiation. XRD measurements were performed at room temperature using a tube voltage of 45 kV, tube current of 40 mA and scan rate of 0.01° 2*θ* s^−1^. The chemical and oxidation states of the catalysts were evaluated by XPS (Thermo Fisher Scientific, ESCALAB250Xi). The oxidation number of Co as the B-site cation was determined using the iodine titration method [27]. A reaction occurs when a sample containing Co is dissolved in an acidic solution containing excess potassium iodide (equation (2.1)). The I_2_ generated when Co is reduced is titrated with sodium thiosulfate (equation (2.2)):


(2.1) $$Co^{\text{2}+x}+(2+x)I^{-}\to CoI_{2}+(x/2)I_{2},$$

(2.2) $$I_{2}+2Na_{2}S_{2}O_{3}\to 2Nal+Na_{2}S_{4}O_{6}.$$

### 2.3. Electrochemical measurements

The ORR and OER activities were evaluated using a rotating disc electrode (RDE). A 5% proton-type Nafion suspension (Sigma-Aldrich) and a 0.1 M KOH aqueous solution were mixed in a 2 : 1 volume ratio. The catalyst inks were prepared by mixing 6.2 mg of the catalyst, 1.2 mg of carbon black (Toka black #4500, Tokai Carbon Co., Japan), 1.21 ml of 2-propanol and 37.5 μl of a 3.3% Nafion suspension. The catalyst ink was stirred with ultrasonication for 30 min. The catalyst ink (30 μl) was dripped onto a glassy-carbon disc electrode (0.1963 cm^2^, model-DENKYOKU, HOKUTO DENKO Corp., Japan). Electrochemical measurements were performed using an RDE rotator (HR-201) in combination with a dual potentiostat (HR-101B) and a function generator (HB-111 (all from HOKUTO DENKO Corp., Japan). A Pt wire electrode and a Hg/HgO electrode (RE-61AP, BAS, Japan) filled with a 0.1 M KOH aqueous solution were used as the counter electrode and reference electrode, respectively. All the electrochemical measurement conditions were maintained at 298 K. In the OER catalyst test, the disc potential was controlled between 0.3 and 0.9 V versus Hg/HgO at a scan rate of 10 mV s^−1^. In the evaluation of the electric double-layer capacity, the disc potential was controlled between −0.88 and 0.31 V versus Hg/HgO at a scan rate of 100 mV s^−1^ in saturated N_2_. In the ORR background measurement, the disc potential was swept from 0.30 to −1.00 V versus Hg/HgO at a scan rate of 10 mV s^−1^ in an N_2_-saturated atmosphere. In the ORR catalyst test, the disc potential was swept from 0.30 to −1.00 V versus Hg/HgO at a scan rate of 10 mV s^−1^, and the disc rotation rates were set at 2500, 1600, 900, 400 and 200 r.p.m. in an O_2_-saturated atmosphere.

### 2.4. Density functional theory calculation

The band structures of LaSrCoO_4_, LaSrCoO_4_-H and LaSrCoO_4_-F were calculated using the Akai KKR software package [28]. For the lattice constants, the value with the most stable total energy was used. The crystal structure of LaSrCoO_4_ is body-centred tetragonal (*a* = 7.231 bohr, *c* = 23.821 bohr). LaSrCoO_4_-H exhibits a body-centred orthorhombic structure (*a* = 7.221 bohr, *b* = 6.716 bohr, *c* = 24.260 bohr). LaSrCoO_4_-F exhibits a body-centred orthorhombic structure (*a* = 7.251 bohr, *b* = 6.743 bohr, *c* = 24.363 bohr). The atomic positions are shown in electronic supplementary material, tables S1−S3. The ewidth, sdftyp, record, bzqlty and pmix were set as 1.7, mjwasa, 2nd, 10 and 0.02, respectively.

## 3. Results and discussion

### 3.1. Characterization of the anion-doped LaSrCoO_4_

**Figure 1.**
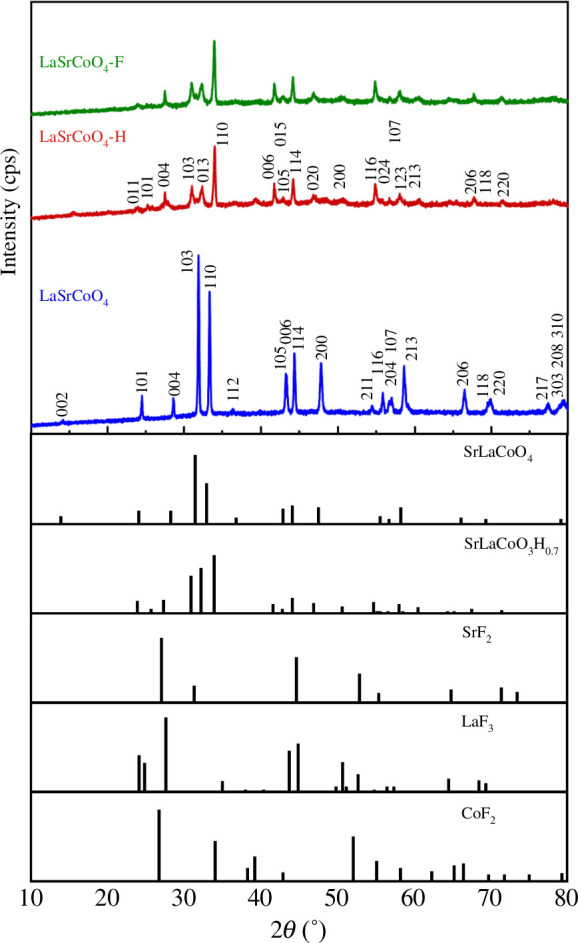
Powder X-ray diffraction patterns for LaSrCoO_4_, LaSrCoO_4_-H and LaSrCoO_4_-F.

The powder XRD patterns for LaSrCoO_4_, LaSrCoO_4_-H and LaSrCoO_4_-F are shown in figure 1. The diffraction peaks of prepared LaSrCoO_4_ were assigned to the tetragonal SrLaCoO_4_ (PDF#83-2410) phase and confirmed as K_2_NiF_4_-type structure. The diffraction peaks of prepared LaSrCoO_4_-H were assigned to the orthorhombic SrLaCoO_3_H_0.7_ (PDF#70-9290) phase. The lack of peaks related to metal fluorides (e.g. SrF_2_, LaF_3_ or CoF_2_) in the XRD patterns indicates that LaSrCoO_4_-F is a single-phase oxyhydride fluoride. When oxide reacts with HF, F^−^ anions do not always undergo substitution at the oxygen sites of the lattice [29]. When oxyhydride reacts with HF, ion exchange between the H^−^ and F^−^ ions occurs, and F^−^ anions are substituted at the H^−^ sites [30]. Therefore, the F^−^ anions are assumed to occupy the H^−^ sites in this experiment. The crystal structure changed from tetragonal to orthorhombic when LaSrCoO_4_ was converted to the oxyhydride, LaSrCoO_3.23_H_0.53_. The crystal structure of LaSrCoO_3.23_H_0.43_F_0.10_ (electronic supplementary material, figure S3) obtained by reacting LaSrCoO_3.23_H_0.53_ and NH_4_F was orthorhombic, and the unit cell volume was approximately the same. We speculated that the exchange of H^−^ (1.48 Å) and F^−^ (1.33 Å) does not affect the size of the unit cell because the ionic radii of H^−^ and F^−^ are similar [30].

**Figure 2.**
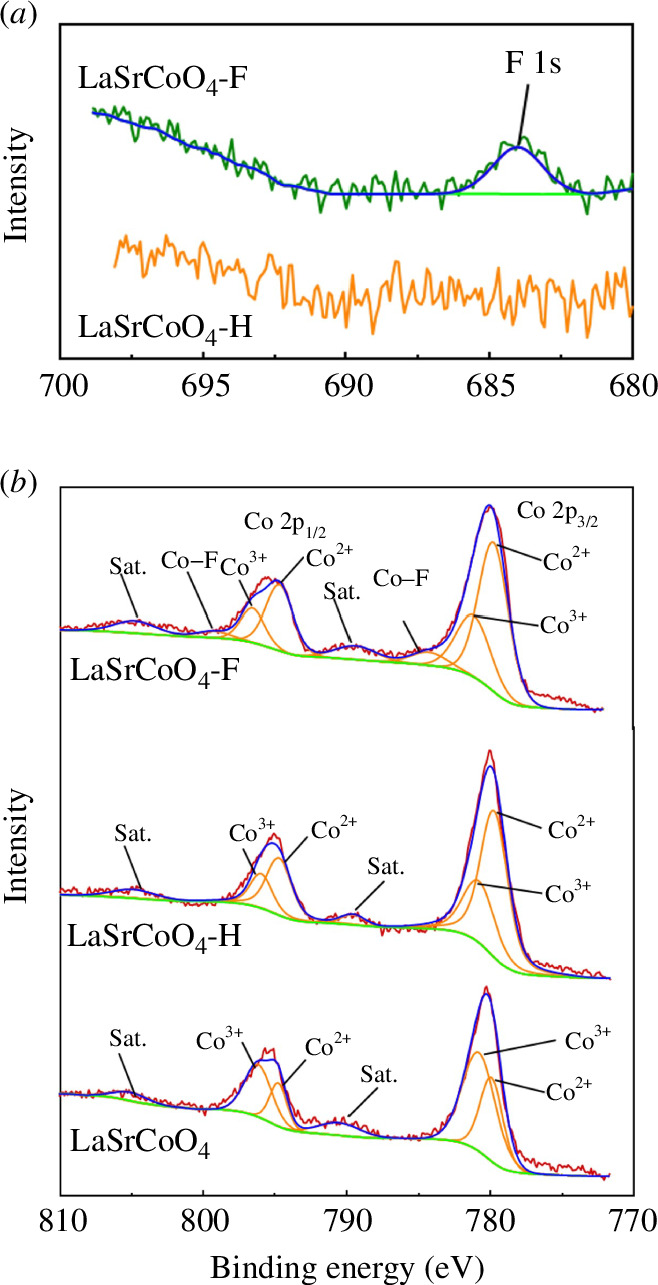
(*a*) F 1s region of the X-ray photoelectron spectroscopy (XPS) spectra of LaSrCoO_4_-H and LaSrCoO_4_-F. (*b*) Co 2p region of the XPS spectra of LaSrCoO_4_, LaSrCoO_4_-H and LaSrCoO_4_-F.

The XPS spectra of LaSrCoO_4_, LaSrCoO_4_-H and LaSrCoO_4_-F are shown in figure 2. A peak attributable to F 1s is observed at 684 eV in the XPS spectrum of LaSrCoO_4_-F (figure 2*a*). This result indicates that F is present in LaSrCoO_4_-F. Peaks attributable to Co 2p_3/2_ and Co 2p_1/2_ were observed in the XPS spectra of LaSrCoO_4_, LaSrCoO_4_-H and LaSrCoO_4_-F (figure 2*b*). The Co species near the surface of the samples exist in a mixed oxidation state of Co^2+^ (779.5, 794.6 eV) and Co^3+^ (780.8, 795.9 eV) [31]. The spectra of LaSrCoO_4_-H and LaSrCoO_4_-F show a larger Co^2+^ peak area ratio than the spectrum of LaSrCoO_4_, indicating that Co^3+^ was reduced during the synthesis of the oxyhydride from the oxide. In addition, the XPS spectrum of LaSrCoO_4_-F displays a peak attributable to Co–F, confirming the presence of F [32]. The valence of Co in the samples was determined using the iodometric titration method. Details of the calculation data are summarized in electronic supplementary material, table S4. The Co valence states in LaSrCoO_4_, LaSrCoO_4_-H and LaSrCoO_4_-F were +2.9, +2.0 and +2.0, respectively (table 1). Thus, Co^3+^ was reduced during the synthesis of the oxyhydride from the oxide, whereas the valence of Co was unchanged during the synthesis of the oxyhydride fluoride from the oxyhydride.

**Table 1.** The Co valence state, average crystallite size, average particle diameter and specific surface area from the Brunauer–Emmett–Teller (SA_BET_) and scanning electron microscopy images (SA_SEM_) of the anion-doped LaSrCoO_4_ catalyst.

sample	Co valence state	average crystallite size (nm)	average particle size (μm)	SA_BET_ (m^2^ g^−1^)	SA_SEM_ (m^2^ g^−1^)
LaSrCoO_4_-F	+2.0	73	0.46	4.1	2.0
LaSrCoO_4_-H	+2.0	61	0.53	3.9	1.8
LaSrCoO_4_	+2.9	111	0.49	2.6	1.9

TG–DTA in air was implemented to validate the anion composition of the compounds. The TG and DTA curves of LaSrCoO_4_, LaSrCoO_4_-H and LaSrCoO_4_-F are shown in electronic supplementary material, figure S1. The TG curve of LaSrCoO_4_ shows no noticeable weight change. The increase in weight observed for LaSrCoO_4_-H and LaSrCoO_4_-F in the temperature range of 220−300°C is attributed to the H^−^ in the lattice reacting with oxygen and desorbing; thus, LaSrCoO_4_-H and LaSrCoO_4_-F are oxidized by oxygen [26]. Since metal fluorides are more thermally stable than the corresponding metal hydrides, the slight increase in the weight of LaSrCoO_4_-F, compared with that for LaSrCoO_4_-H, is attributable to the decrease in the amount of H^−^, owing to its replacement in LaSrCoO_4_-F by F^−^. The compositions of LaSrCoO_4_-H and LaSrCoO_4_-F were determined to be LaSrCoO_3.23_H_0.53_ and LaSrCoO_3.23_H_0.43_F_0.10_ based on the oxidation number of Co and the weight change measured by TG analysis.

**Figure 3.**
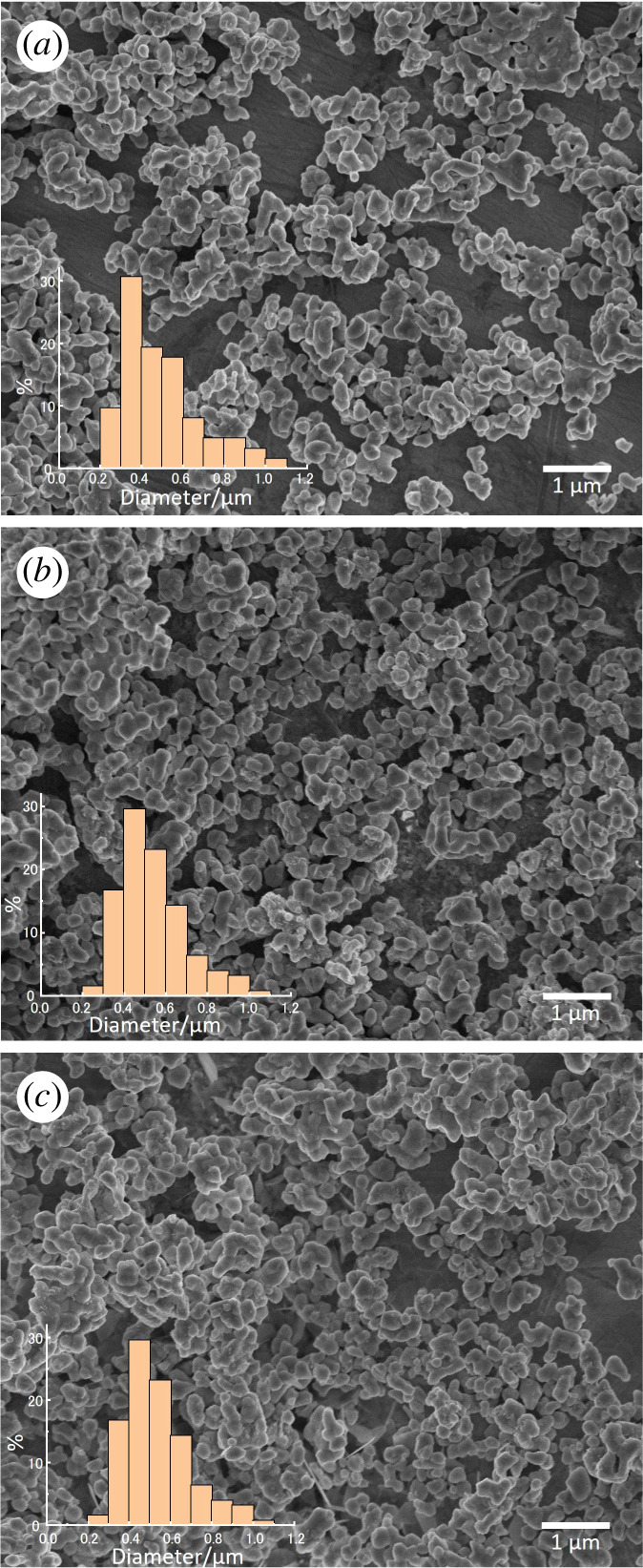
Scanning electron microscopy images and particle size distributions of (*a*) LaSrCoO_4_, (*b*) LaSrCoO_4_-H and (*c*) LaSrCoO_4_-F.

The SEM images of LaSrCoO_4_, LaSrCoO_4_-H and LaSrCoO_4_F are shown in figure 3. The surfaces of the samples show no noticeable differences. The average particle diameters of LaSrCoO_4_, LaSrCoO_3.23_H_0.53_ and LaSrCoO_3.23_H_0.43_F_0.10_ were 0.49, 0.53 and 0.46 μm, respectively (table 1). The lack of a substantial change in the particle size during the synthesis of the mixed-anion compound is attributed to the relatively low-temperature synthesis of LaSrCoO_3.23_H_0.53_ and LaSrCoO_3.23_H_0.43_F_0.10_ (450 and 150°C, respectively), compared with the synthesis temperature of LaSrCoO_4_ (950°C).

The N_2_ adsorption isotherms of LaSrCoO_4_, LaSrCoO_4_-H and LaSrCoO_4_-F at 77 K and the corresponding Brunauer–Emmett–Teller (BET) plot are shown in electronic supplementary material, figure S2. The specific surface areas (SA_BET_) of LaSrCoO_4_, LaSrCoO_3.23_H_0.53_ and LaSrCoO_3.23_H_0.43_F_0.10_ were 2.6, 3.9 and 4.1 m^2^ g^−1^, respectively (table 1). In addition, the specific surface area (SA_SEM_) was calculated using the average particle size obtained from the SEM images (equation (3.1)) [11]:


(3.1) $${SA}_{SEM}=\frac{\Sigma \;4\pi {\left(\frac{d}{2}\right)}^{2}}{\Sigma \;\rho \frac{4}{3}\pi {\left(\frac{d}{2}\right)}^{3}}=\frac{\Sigma \;d^{2}}{\Sigma \;\frac{\rho }{6}d^{3}}=\frac{6}{\rho d_{v/a}},$$

where *ρ* is density and *d*_v/a_ is the volume/average particle size. The SA_SEM_ values of LaSrCoO_4_, LaSrCoO_4_-H and LaSrCoO_4_-F were 1.9, 1.8 and 2.0 m^2^ g^−1^, respectively (table 1). Thus, despite the similar average particle diameters of the samples, the surface areas obtained by the BET method differ from those observed by SEM, indicating that pores likely formed during the synthesis of LaSrCoO_4_-H.

The crystallite sizes (*τ*) of LaSrCoO_4_, LaSrCoO_3.23_H_0.53_ and LaSrCoO_3.23_H_0.43_F_0.10_ were calculated using the Scherrer equation (equation (3.2)) [33]:


(3.2) $$\tau =\frac{K\lambda }{\beta cos\theta },$$

where *β* is the full width at half maximum, *θ* is the Bragg angle, *λ* is the wavelength of the characteristic X-rays used for the measurement (Cu Kα, 1.5418 Å) and *K* is the shape factor (0.89). The *β* and *θ* values associated with the main peak were used for the calculation. The average crystallite sizes of LaSrCoO_4_, LaSrCoO_3.23_H_0.53_ and LaSrCoO_3.23_H_0.43_F_0.10_ were 111, 61 and 73 nm, respectively (table 1). Since the average particle diameter of the compounds was approximately 4−9 times the crystallite diameters, the particles of the obtained compounds were considered to be secondary particles or more. In addition, LaSrCoO_3.23_H_0.53_ and LaSrCoO_3.23_H_0.43_F_0.10_ exhibited smaller crystallite diameters than LaSrCoO_4_; thus, the crystallinity decreased during the synthesis process.

### 3.2. Effect of anion doping on the ORR/OER activity

**Figure 4.**
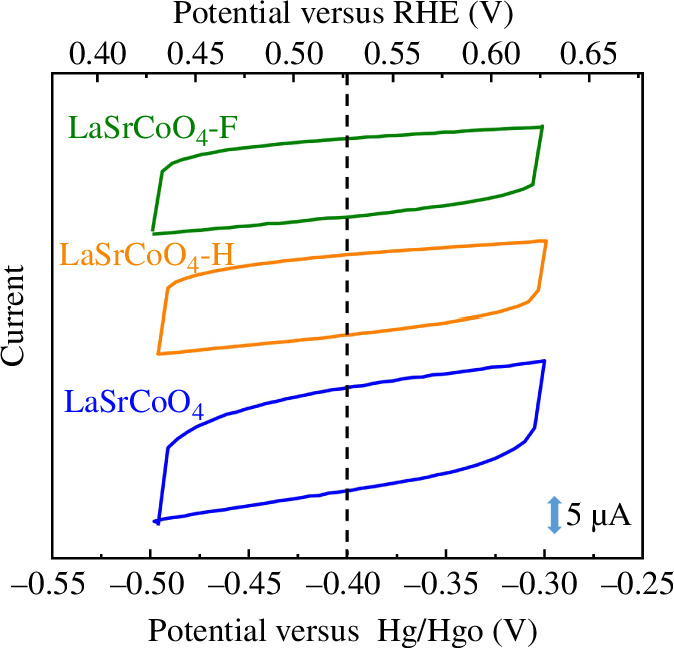
Current–voltage curves of LaSrCoO_4_, LaSrCoO_4_-H and LaSrCoO_4_-F on the glassy-carbon (GC) electrode in N_2_-saturated aqueous 0.1 M KOH solution at a scan rate of 100 mV s^−1^. RHE, reversible hydrogen electrode.


The current–voltage CV curves in figure 4 show the 50th cycle for LaSrCoO_4_, LaSrCoO_4_-H and LaSrCoO_4_-F at the scan rate of 100 mV s^−1^ in a 0.1 M KOH electrolyte saturated with N_2_. The conversion of units fromV versus Hg/HgO (*E*_Hg/HgO_) to V versus the reversible hydrogen electrode (RHE) was calculated using the Nernst equation, activity coefficient and solute concentration, with reference to literature [34]. The parameters used in the calculation are listed in electronic supplementary material, table S5. The electric double-layer capacity was evaluated from the CV curve using (equation 3.3):

(3.3) $$\frac{I_{a}-I_{c}}{2}=C_{dl}\frac{\text{d}E}{\text{d}t},$$

where *I_a_* indicates the oxidation current, *I_c_* is the reduction current, *C*_dl_ is the electric double-layer capacity and d*E/*d*t* is the scan rate. The electric double-layer capacity was determined by averaging 46−50 cycles. The electric double-layer capacities of LaSrCoO_4_, LaSrCoO_4_-H and LaSrCoO_4_-F were 136, 100 and 103 nF, respectively. There is no currently established method for measuring the electrochemical surface area of perovskite oxide. Therefore, in this experiment, the ORR and OER activities were evaluated, and the current value was normalized by the electric double-layer capacity.

**Figure 5.**
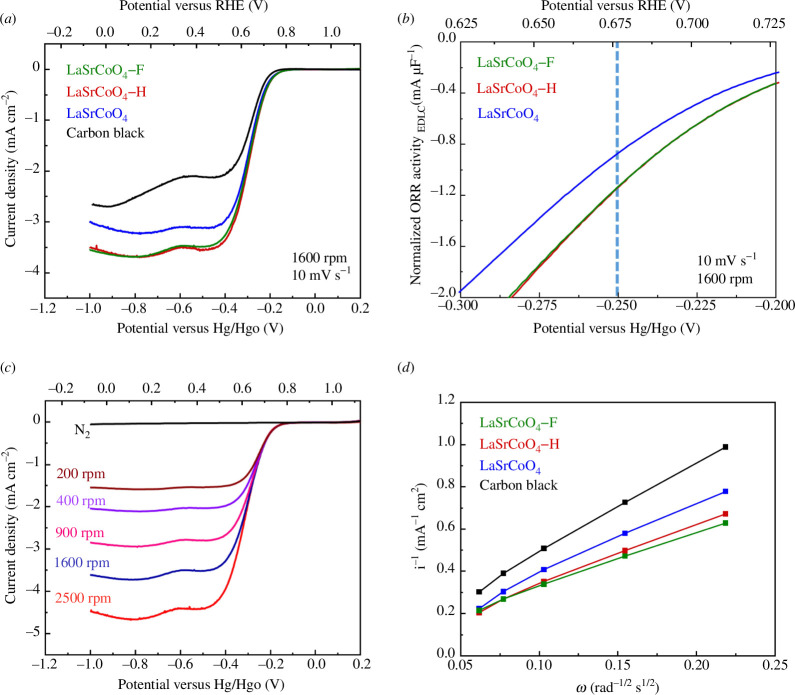
(*a*) Linear sweep voltammetry (LSV) curves of LaSrCoO_4_, LaSrCoO_4_-H, LaSrCoO_4_-F, and carbon black on the glassy carbon electrode in an O_2_-saturated aqueous 0.1 M KOH solution at a scan rate of 10 mV s^−1^. (*b*) LSV curves near the oxygen reduction reaction (ORR) onset potential. (*c*) LSV curves of LaSrCoO_4_-F at a scan rate of 10 mV s^−1^ and rotation rates of 200, 400, 900, 1600 and 2500 r.p.m. (*d*) Koutecky–Levich plots of LaSrCoO_4_, LaSrCoO_4_-H and LaSrCoO_4_-F at a potential of 0.8 V versus Hg/HgO. RHE, reversible hydrogen electrode.


The ORR activities of LaSrCoO_4_, LaSrCoO_4_-H and LaSrCoO_4_-F were evaluated using RDE measurements. Figure 5*a* shows the linear sweep voltammetry (LSV) curves of LaSrCoO_4_, LaSrCoO_4_-H, LaSrCoO_4_-F, and carbon black at a scan rate of 10 mV s^−1^ and rotation rate of 1600 r.p.m. in a 0.1 M KOH electrolyte saturated with O_2_. These LSV curves were subtracted from the background measured under an N_2_ atmosphere. Figure 5*b* shows current–potential curves near the ORR onset potential. The ORR activities at −0.250 V versus Hg/HgO were −0.86,−1.14 and −1.13 mA nF^−1^ for LaSrCoO_4_, LaSrCoO_4_-H and LaSrCoO_4_-F, respectively. LaSrCoO_4_-H and LaSrCoO_4_-F exhibit higher ORR activities than LaSrCoO_4_. Figure 5*c* shows the LSV curve of LaSrCoO_4_-F. The diffusion-limiting current increased from 200 to 2500 r.p.m. In addition, the ORR onset potential remained constant even when the rotation rate changed. Next, the number of electrons involved in the reaction was examined. The ORR in an alkaline electrolyte occurs by two paths: the four-electron reaction (equation (3.4)) and the two-electron reaction (equation (3.5)) [35]. Figure 5*d* shows Koutecky–Levich plots. In the Koutecky–Levich equations (equations (3.6) and (3.7)), *I*_k_ is the reaction current, *ω* is the rotation rate of the electrode, *n* is the number of reaction electrons for 1 mol of oxygen, *F* is the Faraday constant (96 485 C mol^−1^), *D*_0_ is the diffusion coefficient of oxygen in the 0.1 M KOH electrolyte (1.9 × 10^−5^ cm^2^ s^−1^), *v* is the kinetic viscosity (0.01 cm^2^ s^−1^) and *C*_0_ is the oxygen concentration [36]. The number of reaction electrons (*n*) was 2.5, 3.1 and 3.5 for LaSrCoO_4_, LaSrCoO_4_-H and LaSrCoO_4_-F, respectively. From this result, it is evident that the ORR mechanism of LaSrCoO_4_ is dominated by the two-electron reaction, that of LaSrCoO_4_-H involves competing a two-electron reaction and a four-electron reaction, and that of LaSrCoO_4_-F is dominated by a four-electron reaction. In the case of two-electron reactions, the oxygen adsorbed on the active site is reduced without dissociation, to form hydrogen peroxide. On the other hand, in the case of four-electron reactions, the bonds between the oxygen atoms are dissociated during the reaction process. The average number of reaction electrons was increased by doping hydride and fluorine into LaSrCoO_4_, indicating that the oxygen molecules were more easily dissociated at the reaction sites on the catalyst surface. The electronic state of cobalt was changed by the substitution of oxygen on the surface of LaSrCoO_4_ for hydride or fluorine by topochemical reactions. It is considered that the change in the electronic state of cobalt, which was directly bound to the anions, improved the adsorption and dissociation ability of oxygen, thereby increasing the number of reaction sites where four-electron reactions proceeded on the catalyst:

(3.4) $$O_{2}\;\;+\;\;2H_{2}O\;\;+\;\;4e^{-}\;\to \;\;4OH^{-},$$

(3.5) $$O_{2}+H_{2}O+2e^{-}\to OH^{-}+HO_{2}^{-},$$

(3.6) $$\frac{1}{I}=\frac{1}{I_{k}}+\;\frac{1}{B\sqrt{\omega }},$$

(3.7) $$B=0.62nF{\left(D_{0}\right)}^{2/3}v^{-1/6}C_{0}.$$

It has been reported that the electronic state of the B-site cation is important in evaluating the activity of perovskite oxide catalysts [10]. As mentioned above, the Co oxidation number was 2.9, 2.0 and 2.0 for LaSrCoO_4_, LaSrCoO_4_-H and LaSrCoO_4_-F, respectively. In addition, the ORR activity decreased in the following order: LaSrCoO_4_-H ≈ LaSrCoO_4_-F > LaSrCoO_4_. The number of reaction electrons decreased in the order of LaSrCoO_4_-F > LaSrCoO_4_-H > LaSrCoO_4_. Therefore, the rate-determining step of the ORR on the perovskite is said to be the electron transfer from the e_g_ orbital of the B-site cation to the O π^*^ orbital [10]. Suntivich *et al*. employed a volcanic plot to illustrate the relationship between the ORR activity and the number of d electrons at the B-site. They revealed that the activity is high when the number of d electrons is 3.7 or 6.7 [10]. The number of d electrons calculated from the oxidation number of Co was 6.1, 7.0 and 7.0 for LaSrCoO_4_, LaSrCoO_4_-H and LaSrCoO_4_-F, respectively. The electron transfer efficiency from the B-site cation e_g_ orbital to the O π^*^ orbital increased. Correspondingly, the ORR activity of LaSrCoO_4_-H and LaSrCoO_4_-F improved because their d electron count was close to 6.7.

In this experiment, the number of electrons involved in the reaction was 2.5, 3.1 and 3.5 for LaSrCoO_4_, LaSrCoO_4_-H and LaSrCoO_4_-F, respectively. The number of electrons for LaSrCoO_4_-F was the closest to the value of 4. Zhao *et al*. predicted that the ORR activity of F-doped catalysts was low because of the relationship between the electronegativity and electron-affinity properties of the catalyst. However, here, only the F-doped catalyst exhibited a high ORR activity [37]. In addition, the reaction mechanism with the F-doped catalysts tended to be dominated by the four-electron reaction. This is attributed to the high reactivity of F and the surrounding electronic structure [38,39]. Thus, the number of reaction electrons of LaSrCoO_4_-F is close to four probably because of the characteristics of F.

The OER activities of LaSrCoO_4_, LaSrCoO_4_-H and LaSrCoO_4_-F were evaluated by RDE measurements. The OER in alkaline electrolytes proceeds by a four-electron reaction (equation (3.8)):


(3.8) $$4OH^{-}\;\;\to \;\;O_{2}\;\;+\;\;2H_{2}O\;\;+\;\;4e^{-}.$$

**Figure 6.**
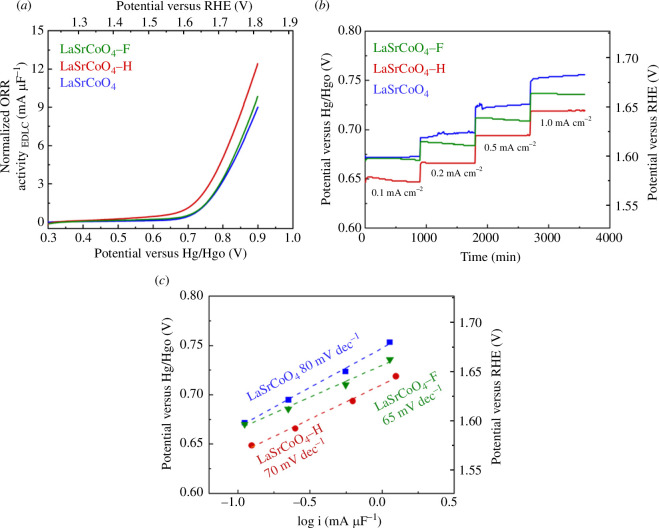
(*a*) Current–potential (*I*–*V*) curves of LaSrCoO_4_, LaSrCoO_4_-H and LaSrCoO_4_-F on the GC electrode in an O_2_-saturated 0.1 M KOH electrolyte at a scan rate of 10 mV s^−1^ with electrode rotation rate at 1600 r.p.m. (*b*) Chronopotentiometry measurement results. Measured for 15 min at current densities of 0.1, 0.2, 0.5 and 1.0 mA cm^−2^, separately. (*c*) Tafel plots and Tafel slopes of the catalysts. Currents were normalized to the electric double-layer capacity. ORR, oxygen reduction reaction; RHE, reversible hydrogen electrode.

Figure 6(*a*) shows the current–potential curves. The current value was normalized by the electric double-layer capacity. The OER activities at 0.82 V versus Hg/HgO (1.75 V versus RHE) were 4.2, 6.5 and 4.5 mA μF^−1^ for LaSrCoO_4_, LaSrCoO_4_-H and LaSrCoO_4_-F, respectively. The OER activity decreased in the order of LaSrCoO_4_-H > LaSrCoO_4_-F > LaSrCoO_4_. In addition, figure 6(*b*) shows the chronopotentiometry measurement results. Figure 6(*c*) shows Tafel plots with slopes of 80, 70 and 65 mV decade^−1^ for LaSrCoO_4_, LaSrCoO_4_-H and LaSrCoO_4_-F, respectively. These values are comparable to previously reported Tafel slopes for La, Sr and Co-based perovskite catalysts (LaCoO_3_: 74 mV decade^−1^, LaSrCoO_4_: 88 mV decade^−1^, Ba_0.5_Sr_0.5_Co_0.8_Fe_0.2_O_3−*δ*_: 72 mV decade^−1^ [19], Cl_0.02_-LaCoO_3_: 76.2 mV decade^−1^ [40], SrCoO_2.85−*δ*_F_0.15_: 60 mV decade^−1^ [41], Sr_2_CoO_3_Cl: 60 mV decade^−1^, Sr_3_Co_2_O_5_Cl_2_: 62 mV decade^-1^ [19] and La_0.5_Ba_0.25_Sr_0.25_CoO_2.9–*δ*_F_0.1_: 113 mV decade^-1^ [42]). This indicates that hydride- and fluorine-doped LaSrCoO_4_ compounds are competitive with other perovskite-based catalysts as an OER catalysts.

**Figure 7.**
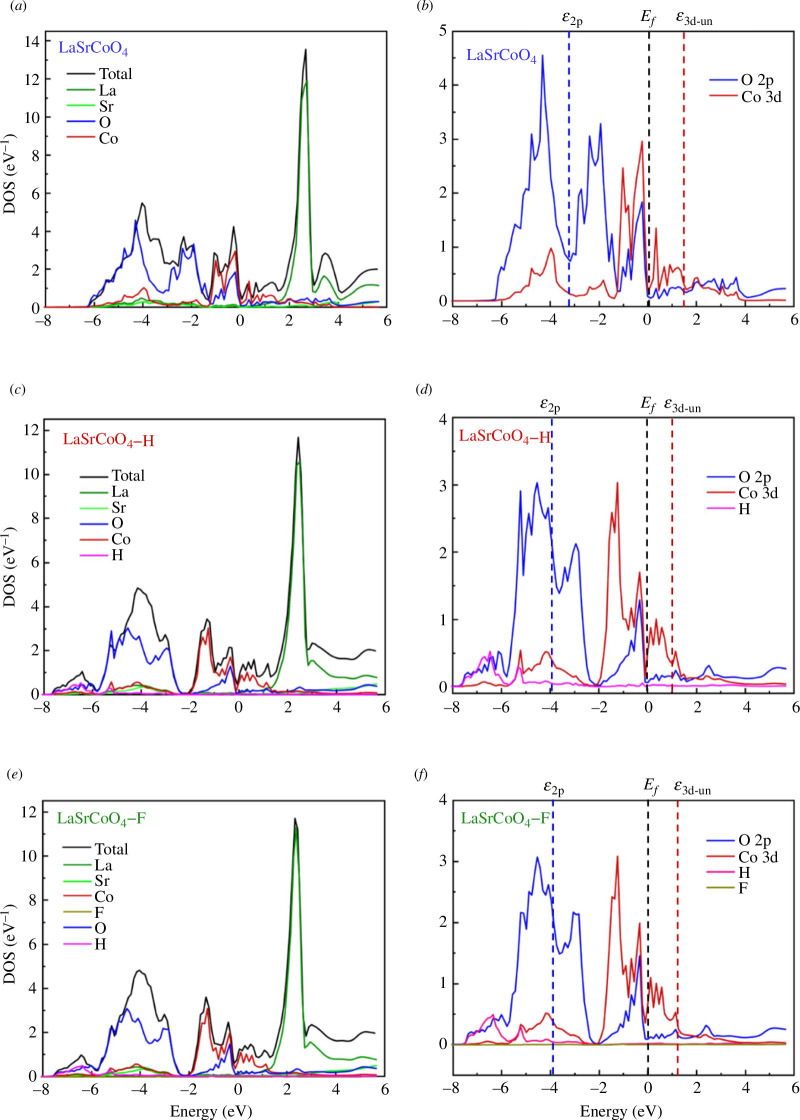
Total (per formula unit) density of states (DOS) of (*a*) LaSrCoO_4_, (*c*) LaSrCoO_4_-H and (*e*) LaSrCoO_4_-F; Co 3d (per formula unit) and O 2p (per formula unit) density of states of (*b*) LaSrCoO_4_, (*d*) LaSrCoO_4_-H and (*f*) LaSrCoO_4_-F obtained by density functional theory calculations.


Previous studies have reported that the OER activity of perovskite oxides is related to the O 2p and 3d bands of the B-site cation states [12,13]. Therefore, the band structures of LaSrCoO_4_, LaSrCoO_4_-H and LaSrCoO_4_-F were analysed using DFT. As shown in figure 7, La 4f exists as a sharp peak near 2.5 eV, whereas La 5d and Sr 4d are distributed at higher energies. Co 3d exists near the Fermi level. LaSrCoO_4_ features the O 2p peak, which is distributed from 0.0 to −6.2 eV. Conversely, LaSrCoO_4_-H has a wide distribution of O 2p from 0.0 to −7.8 eV. LaSrCoO_4_-F produced a low-intensity F 2p peak near −0.9 eV and a sharp peak near 10 eV. The O 2p band centre (equation (3.9)) and the Co 3d unoccupied orbital band centre (equation (3.10)) were determined using a weighted average [43,44]. In the equations, *ε*_2p_ is the O 2p band centre, *E*_f_ is the Fermi level and *ε*_3d-un_ is the Co 3d unoccupied orbital band centre. In addition, the charge-transfer energy (*Δ*) was determined using equation (3.11):


(3.9) $$\varepsilon_{2p}=\frac{\int_{-\infty }^{E_{f}}Ef_{2p}\left(E\right)\text{d}E}{\int_{-\infty }^{E_{f}}f_{2p}\left(E\right)\text{d}E},$$

(3.10) $$\varepsilon_{3d\text{-}un}=\frac{\int_{E_{f}}^{E_{max}}Ef_{3d}\left(E\right)dE}{\int_{E_{f}}^{E_{max}}f_{3d}\left(E\right)dE},$$

(3.11) $$\Delta =\varepsilon_{3d\text{-}un}-\varepsilon_{2p}.$$

**Figure 8.**
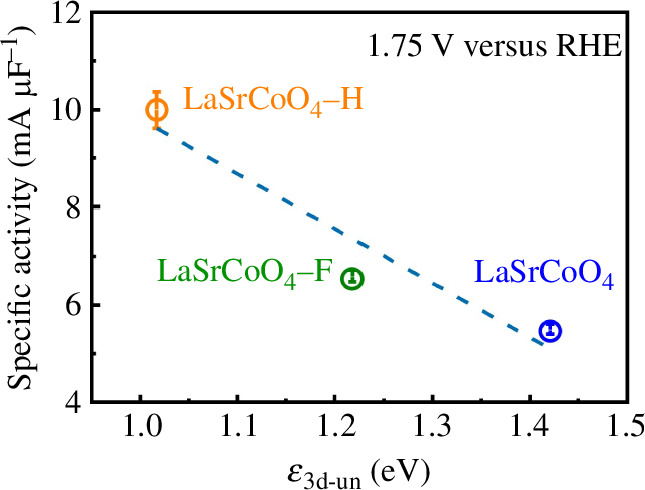
Relationship between the normalized oxygen evolution reaction activity and the Co 3d unoccupied band centre. RHE, reversible hydrogen electrode.

Table 2 shows the O 2p band centre, Co 3d unoccupied band centre and charge-transfer energy. It has been reported that the OER activity of the O 2p band centre closer to the Fermi level is high [12]. However, in this experiment, the O 2p band centres were −3.32, −3.96 and −3.92 eV for LaSrCoO_4_, LaSrCoO_4_-H and LaSrCoO_4_-F, respectively. The O 2p band centre of the anion-added catalyst is located far from the Fermi level. The band exists at −4.9 to −7.6 eV. Since the orbitals of the Co 3d, O 2p and H anion are hybridized [45], it is not reasonable to evaluate the OER activity at the O 2p band centre. In addition, for all three catalysts, the top positions of the O 2p band near the Fermi level are not significantly different. Figure 8 shows the relationship between the normalized OER activity and the Co 3d unoccupied band centre. It is found that the smaller the Co *ε*_3d-un_, the higher the OER activity. Therefore, the rate-determining step of the OER is reported to be electron transfer from the adsorbed oxygen species to the B-site cation [11,46]. In this case, the e_g_ orbitals ( ${{\text{d}}_{Z}}^{2}$ , ${\text{d}}_{x^{2}-y^{2}}$ ) of the B-site cation are involved in the exchange electron. The position of the 3d unoccupied orbital is related to the energy barrier for the charge-transfer process at the OER rate-determining step [47]. In this experiment, since Co *ε*_3d-un_ is close to the Fermi level and the energy barrier for charge-transfer is reduced, the OER activity was improved.

**Table 2.** Calculated O 2p-band centres (*ε*_2p_), unoccupied Co 3d band centres (*ε*_3d-un_) and charge-transfer energy (*Δ*) of LaSrCoO_4_, LaSrCoO_4_-H and LaSrCoO_4_-F.

sample	*ε*_2p_ (eV)	*ε*_3d-un_ (eV)	*Δ* (eV)
LaSrCoO_4_	−3.32	1.42	4.74
LaSrCoO_4_-H	−3.96	1.02	4.97
LaSrCoO_4_-F	−3.92	1.22	5.14

Finally, XRD measurements of the catalyst after the electrochemical reaction were performed to study the stability of the anion-doped catalyst. Since the amount of catalyst used in the electrochemical reaction was small, the measurement was performed with the catalyst attached to a glassy carbon (GC) electrode. The XRD results showed that the diffraction peaks attributed to the LaSrCoO_4_-H or LaSrCoO_4_-F were observed even after ORR and OER (electronic supplementary material, figure S4). Thus, it was confirmed that the anion-doped compounds used as catalysts were not significantly decomposed during the electrochemical reaction. XPS spectra were also measured to investigate the stability of the anions doped to LaSrCoO_4_. XPS measurements were performed on the catalyst after the electrochemical reaction in the state of catalyst ink, which was detached from the GC electrode. Comparison of the Co^2+^/Co^3+^ area ratios from the Co 2p_3/2_ peaks in the XPS spectra showed that LaSrCoO_4_-H has a larger change in area ratio after ORR and OER (electronic supplementary material, figures S5–S7). This result suggests that LaSrCoO_4_-H is more unstable than other catalysts. On the other hand, comparison of the F 1*s* peak of LaSrCoO_4_-F showed that the peak attributed to Co-F was still observed after the reaction (electronic supplementary material, figure S8). Thus, the F added to LaSrCoO_4_-F was stable during the electrochemical measurements. These results suggest that hydride-doped LaSrCoO_4_ is relatively unstable to electrochemical reactions, and further fluorine addition effectively improves its stability.

## 4. Conclusion

In this study, a novel oxyhydride fluoride was synthesized using the corresponding oxyhydride as a precursor. XPS analysis showed that Co–F bonds were formed via the reaction of LaSrCoO_4_ with NH_4_F. The anion composition of LaSrCoO_4_-F was determined to be LaSrCoO_3.23_H_0.43_F_0.10_ based on the iodometric analysis of the Co oxidation number and the TG analysis of the anion composition. In addition, Rietveld refinement of the powder XRD data for LaSrCoO_3.23_H_0.43_F_0.10_ revealed that the crystal structure of LaSrCoO_3.23_H_0.43_F_0.10_ exhibits *Immm* orthorhombic space symmetry, with lattice parameters of *a* = 3.629(8), *b* = 3.878(8) and *c* = 12.948(7) Å. During the synthesis of oxyhydride and oxyhydride fluoride from oxides, almost no change in the particle diameter or surface area was observed. The valence of Co and the crystal structure changed during the formation of the oxyhydride from the oxide but did not change when the oxyhydride fluoride was prepared from the oxyhydride. Thus, such a low-temperature topochemical reaction may be an effective method for synthesizing novel mixed-anion compounds.

The effect of anion doping on the ORR/OER activity of LaSrCoO_4_ was also investigated. The ORR activity of the anion-added perovskites was relatively high, and the catalytic mechanism of the newly synthesized LaSrCoO_4_-F was dominated by a four-electron reaction. The OER activity of LaSrCoO_4_-H was the highest in this experiment. A correlation was found between the OER activity and the Co 3d unoccupied orbital band centre by DFT calculations. The results confirm that anion doping, as well as metal-site substitution, is effective in enhancing the ORR and OER activities of perovskite electrocatalysts.

## Data Availability

All data (i.e. atomic parameters of catalysts, calculation data of cobalt valence number and Hg/HgO electrode potential, thermogravimetry curves, nitrogen adsorption isotherms of catalysts, crystal structure of LaSrCoO4-F, XRD patterns and XPS spectra of the catalysts after ORR or OER) are provided in the electronic supplementary material accompanying this article.
